# Distinct Prognostic and Immunological Roles of ETS1 and ETS2: A Pan-Cancer Analysis

**DOI:** 10.1155/2023/4343350

**Published:** 2023-01-31

**Authors:** Yajun Ren, Bing Chen, Meng Zhang

**Affiliations:** ^1^Department of Gastroenterology, The First Affiliated Hospital of Zhengzhou University, Zhengzhou 450052, China; ^2^Department of Clinical Laboratory, The First Affiliated Hospital of Zhengzhou University, Zhengzhou 450052, China; ^3^Key Clinical Laboratory of Henan Province, The First Affiliated Hospital of Zhengzhou University, Zhengzhou 450052, China

## Abstract

**Objective:**

ETS1 and ETS2, the main ETS family of transcription factors, have been found to act as downstream effectors of the RAS/MAPK pathway. This study explores the expression and prognostic values of ETS1 and ETS2 across cancers. We also aimed to explore the significance of ETS1 and ETS2 expression in normal immune cells with relation to tumorigenesis.

**Methods:**

The expression of ETS1 and ETS2 was examined in the HPA and GEPIA2 databases. The KM plotter was applied to examine prognostic value of ETS1 and ETS2. Correlation between ETS1/ETS2 and infiltrating immune cells and immune checkpoints was assessed using TIMER2.0. The mutation landscape of ETS1/ETS2 was explored using the cBioPortal. STRING and GEPIA2 were used to screen ETS1/ETS2 binding and correlated genes. Enrichr was applied to perform GO and KEGG enrichment analyses.

**Results:**

ETS1 showed enhanced expression in lymphoid tissue, while ETS2 showed low tissue specificity. ETS1 was increased in 12 and decreased in 6 cancers, while ETS2 was increased in 4 and decreased in 13 cancers. Both ETS1 and ETS2 were favorable prognostic markers in LIHC and KIRC, while they showed different prognostic roles in more cancers. ETS1 showed stronger correlation with several infiltrating immune cells and immune checkpoints compared with ETS2. Both ETS1 and ETS2 harbored low mutation ratio. ETS1 interacting and correlated genes were enriched in GO terms in response to cadmium ion and response to oxidative stress, while those of ETS2 were enriched in transcription regulation.

**Conclusion:**

ETS1 and ETS2 showed different patterns in expression, prognostic values, correlation with immune infiltrating, and immune checkpoints. ETS1 and ETS2 play distinct roles across cancer.

## 1. Introduction

The conserved ETS DNA-binding GGAA/T motif is the common feature of the ETS family of transcription factors, and ETS1 and ETS2 are the main members, which act as downstream targets of the RAS/MAPK pathway [1]. ETS transcription factors promote tumorigenesis via regulating the transcription of numerous genes through this ETS DNA-binding domain [2]. Aberrant activation of these family members enhances tumorigenesis via a variety of mechanisms, including DNA damage and genome instability, self-renewal and lineage specification, epigenetics, and metabolism. [3]. ETS1 and ETS2 are also involved in tumor suppression under some situations [4].

Both ETS1 and ETS2 were necessary for the RAS-oncogenic transformation revealed by ETS1/ETS2 double knockout mice [5]. However, under some situations, ETS1 and ETS2 play different or opposite roles. ETS1 but not ETS2 was found to inhibit antibody-secreting cell differentiation [6]. ETS2 was associated with proliferation and cellular activation; however, ETS1 was mainly expressed in quiescent state of human T cells [7]. ETS1 and ETS2 oppositely regulated the expression of genes for cell migration due to transcriptional attenuation and binding site competition. ETS2 may act to switch oncogenic role to tumor suppressive role [8]. These studies suggest different roles of ETS1 and ETS2 under different situations.

We performed a pan-cancer integrated bioinformatic analysis to explore the prognostic potential and immune features of ETS1 and ETS2 across cancers. We also aimed to explore the significance of ETS1 and ETS2 expression in normal immune cells with relation to tumorigenesis.

## 2. Methods

### 2.1. Expression of ETS1 and ETS2

HPA (https://www.proteinatlas.org/) was applied to examine the expression of ETS1 and ETS2 in normal human tissues and cells. GEPIA2 (http://gepia2.cancer-pku.cn/) was equipped to compare the expression difference between TCGA tumors and normal samples combing TCGA and GTEx normal tissues using ANOVA [9].

### 2.2. Prognostic Potential Analysis of ETS1 and ETS2

The KM plotter (http://kmplot.com/analysis/) was applied to examine the prognostic potential of ETS1 and ETS2 across cancer using the Cox-Mantel (log rank) test [10].

### 2.3. Immune Infiltration and Immune Checkpoint Analysis

Correlation between ETS1/ETS2 and immune infiltration and immune checkpoint genes was performed using TIMER2.0 (http://timer.cistrome.org/) based on Pearson's correlation method [11].

### 2.4. Mutation Landscape of ETS1 and ETS2

A pan-cancer mutation landscape of ETS1 and ETS2 was examined using cBioPortal (http://www.cbioportal.org), including amplification, deep deletion, mutation, and multiple alterations [12].

### 2.5. GO and KEGG Enrichment Analyses

The interacting genes of ETS1 and ETS2 were screened on STRING (https://string-db.org). GEPIA2 was applied to screen the most correlated genes of ETS1 and ETS2 in TCGA tumors. The GO and KEGG enrichment analyses were performed with Enrichr (https://maayanlab.cloud/Enrichr/) [13].

## 3. Results

### 3.1. Expression Characteristics of ETS1 and ETS2 in Normal Tissues and Cancers

**Figure 1:**
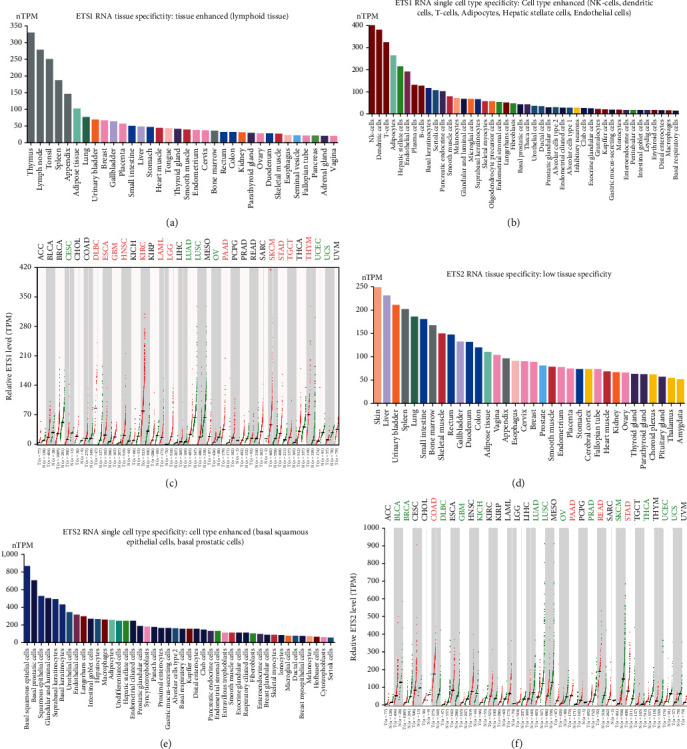
Expression characteristics of ETS1 and ETS2 in normal tissues and cancers. (a, d) The expression of ETS1 and ETS2 in normal human tissues. (a) The tissue expression of ETS1 and (d) the tissue expression of ETS2. (b, e) The expression of ETS1 and ETS2 in normal human cells. (b) The cell expression of ETS1 and (e) the cell expression of ETS2. (c, f) A pan-cancer analysis of the expression of ETS1 and ETS2. Nontumor samples consisted of samples from TCGA and GTEx databases. Abbreviations colored in red represents increased expression in cancers, while green color indicates decreased expression.

The expression characteristics of ETS1 and ETS1 in normal human tissues and cells were explored in the HPA database. ETS1 showed enhanced expression in lymphoid tissue, while ETS2 showed low tissue specificity (Figure 1(a)). ETS2 showed relative high expression in rectum, duodenum, and colon. The cellular expression analysis showed that ETS1 was enhanced in NK cells, dendritic cells, and T cells, while ETS2 wan enhanced in basal squamous epithelial and prostatic cells with low immune cell specificity (Figure 1(b)). Then the expression of ETS1 and ETS2 between tumor and normal samples was compared across 33 types of tumors included in the TCGA database (Figure 1(c)). ETS1 was increased in esophageal carcinoma (ESCA), lymphoid neoplasm diffuse large B-cell lymphoma (DLBC), glioblastoma multiforme (GBM), head and neck squamous cell carcinoma (HNSC), acute myeloid leukemia (LAML), kidney renal clear cell carcinoma (KIRC), brain lower grade glioma (LGG), skin cutaneous melanoma (SKCM), pancreatic adenocarcinoma (PAAD), stomach adenocarcinoma (STAD), testicular germ cell tumors (TGCT), and thymoma (THYM), while reduced in lung adenocarcinoma (LUAD), cervical squamous cell carcinoma and endocervical adenocarcinoma (CESC), lung squamous cell carcinoma (LUSC), uterine corpus endometrial carcinoma (UCEC), ovarian serous cystadenocarcinoma (OV), and uterine carcinosarcoma (UCS). ETS2 was remarkably increased in colon adenocarcinoma (COAD), PAAD, rectum adenocarcinoma (READ), and STAD, while decreased in breast invasive carcinoma (BRCA), bladder urothelial carcinoma (BLCA), DLBC, GBM, kidney chromophobe (KICH), LUAD, LUSC, prostate adenocarcinoma (PRAD), SKCM, thyroid carcinoma (THCA), UCEC, and UCS.

Both ETS1 and ETS2 were increased in PAAD and STAD, while both were decreased in LUAD, LUSC, OV, UCEC, and UCS. ETS2 seemed to be increased mainly in tumors of digestive system including COAD, READ, and STAD, while decreased mainly in tumors of urogenital system including BLCA, KICH, and PRAD. ETS1 dysregulation did not show any tissue specificity.

### 3.2. Prognostic Values of ETS1 and ETS2 across Cancers

**Figure 2:**
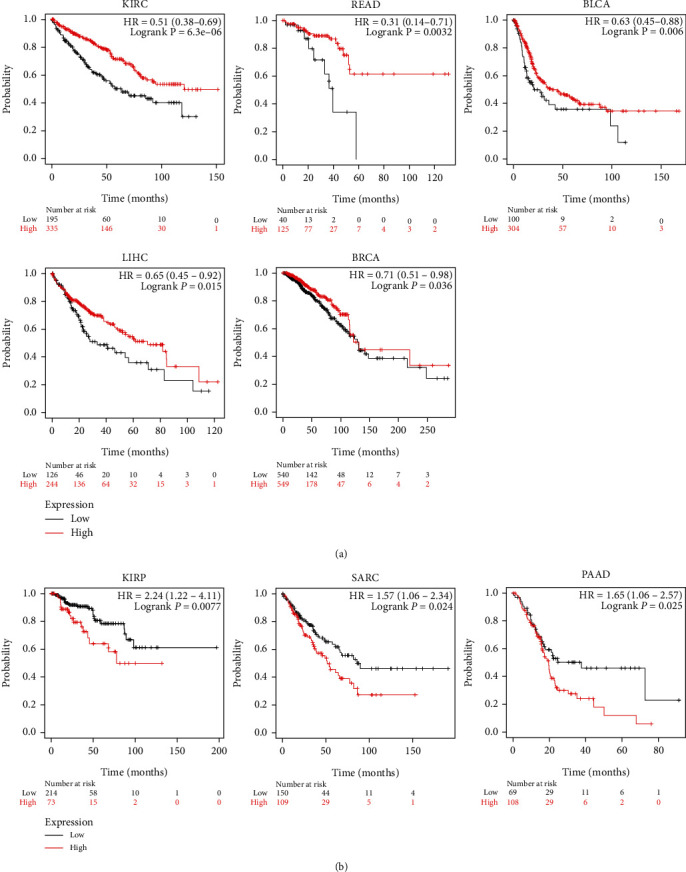
Prognostic value of ETS1 in cancers. (a) Survival curves of KIRC, READ, BLCA, LIHC, and BRCA. (b) Survival curves of KIRP, SARC, and PAAD. OS: overall survival; HR: hazard ratio.

A pan-cancer prognostic analysis of ETS1 and ETS2 was assessed using the KM plotter. High ETS1 expression correlated with long OS in KIRC (HR = 0.51, 95% CI: 0.38-0.69), READ (HR = 0.31, 95% CI: 0.14-0.71), BLCA (HR = 0.63, 95% CI: 0.45-0.88), liver hepatocellular carcinoma (LIHC; HR = 0.65, 95% CI: 0.45-0.2), and BRCA (HR = 0.71, 95% CI: 0.51-0.98) (Figure 2(a)). However, high ETS1 level was associated with short OS in KIRP (HR = 2.24, 95% CI: 1.22-4.11), sarcoma (SARC; HR = 1.57, 95% CI: 106-2.34), and PAAD (HR = 1.65, 95% CI: 1.06-2.57) (Figure 2(b)).

**Figure 3:**
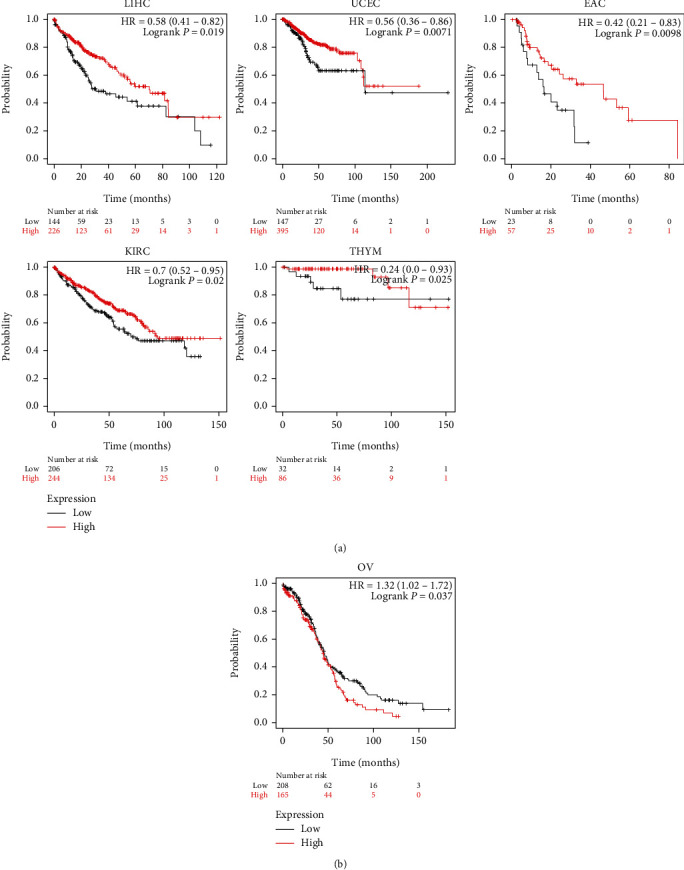
Prognostic value of ETS2 in cancers. (a) Survival curves of LIHC, UCEC, EAC, KIRC, and THYM. (b) Survival curves of OV.

High ETS2 expression was associated with long OS in LIHC (HR = 0.58, 95% CI: 0.41-0.82), UCEC (HR = 0.56, 95% CI: 0.36-0.86), esophageal adenocarcinoma (EAC; HR = 0.42, 95% CI: 0.21-0.83), KIRC (HR = 0.75, 95% CI: 0.52-0.95), and THYM (HR = 0.24, 95% CI: 0.05-0.93) (Figure 3(a)). However, high ETS2 expression was associated with short OS in OV (HR = 1.32, 95% CI: 1.02-1.72) (Figure 3(b)). ETS2 plays different prognostic roles in OV.

Taken together, both ETS1 and ETS2 were positively correlated with long OS in KIRC and LIHC. However, ETS1 and ETS2 showed different prognostic values across several cancers.

### 3.3. Correlation between ETS1/ETS2 and Immune Infiltration

**Figure 4:**
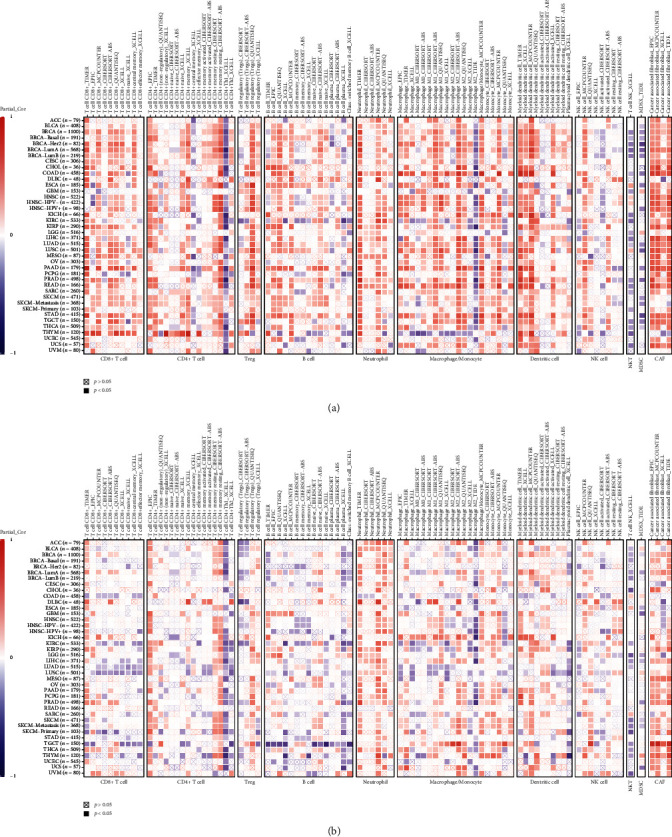
Correlation between expression of ETS1/ETS2 and immune infiltration. (a) The correlations between ETS1 expression and infiltrating immune cells across multiple cancers. (b) The correlations between ETS2 expression and infiltrating immune cells across multiple cancers. Only *p* > 0.05 was labeled with ⊠ in the plot.

Correlation between ETS1/ETS2 and immune infiltration was analyzed using TIMER 2.0. In most tumors, ETS1 showed strong correlation with several immune cells (Figure 4(a)), including T cells, B cells, macrophages/monocytes, cancer-associated fibroblasts (CAFs), and DCs. However, in THYM, ETS1 showed negatively correlation with B cells, macrophages/monocytes, CAFs, and NK cells, while positively associated with DCs and T cells.

Compared to ETS1, the correlation coefficient between ETS2 and infiltrating immune cells was lower. ETS2 showed moderate positive correlation with T cells, B cells, DCs, and CAFs (Figure 4(b)). However, in TGCT, ETS2 was negatively associated with T cells and B cells, while positively correlated with macrophages/monocytes, DCs, and CAFs (Figure 4(b)).

### 3.4. Correlation between Immune Checkpoint Genes and ETS1/ETS2

**Figure 5:**
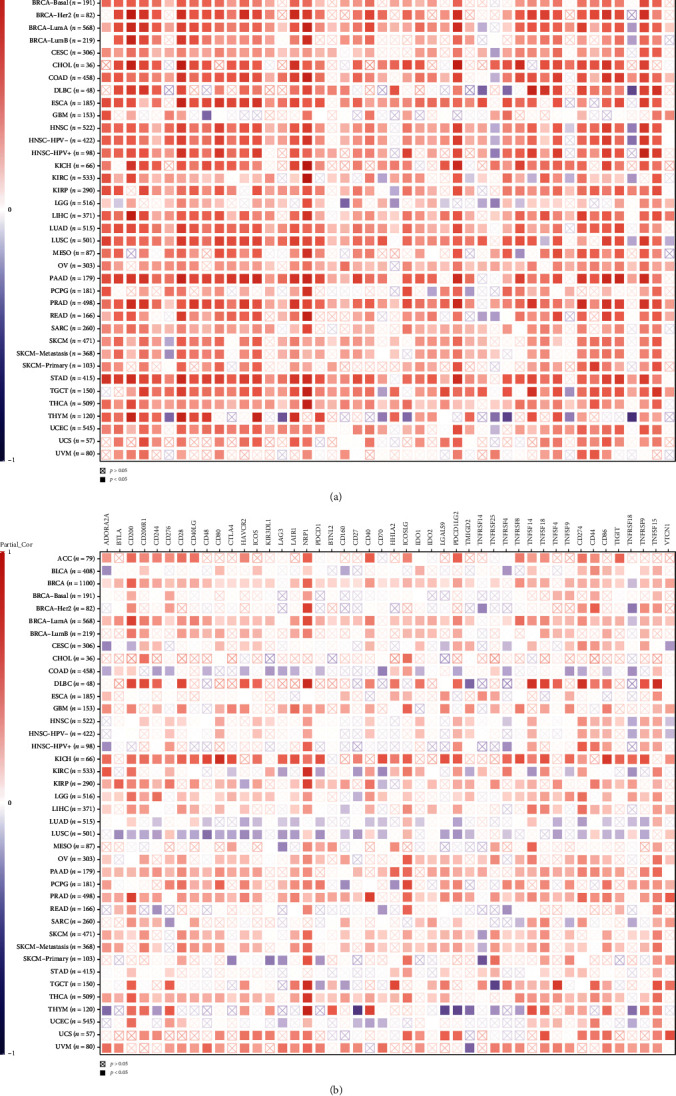
Correlation between immune checkpoint genes and ETS1/ETS2. (a) Correlation between ETS1 and immune checkpoint genes in multiple cancers. (b) Correlation between ETS1 and immune checkpoint genes in multiple cancers. Only *p* > 0.05 was labeled with ⊠ in the plot.

ETS1 showed significant positive correlation with numerous immune checkpoint genes across multiple cancers (Figure 5(a)), including CD200, CD28, LAIR1, NRP1, PDCD1LG2, CD274, TNFRSF9, and TNFSF15. However, in THYM, ETS1 was negatively correlated with CD276, LAG3, CD70, ICOSLG, TNFRSF25, TNFRSF4, TNFSF9, and TNFRSF18. THYM showed unique correlation between immune checkpoint genes and ETS1 expression compared with other types of cancers.

ETS2 showed weaker correlation with immune checkpoint genes compared with ETS1 (Figure 5(b)). ETS2 was positively correlated with CD200, NRP1, ICOSLG, and TNFSF15 across several cancers. Negative correlation between ETS2 and immune checkpoint genes was observed in multiple cancers, including COAD, LUAD, and LUSC.

### 3.5. Mutation Landscape of ETS1 and ETS2 in across Cancers

**Figure 6:**
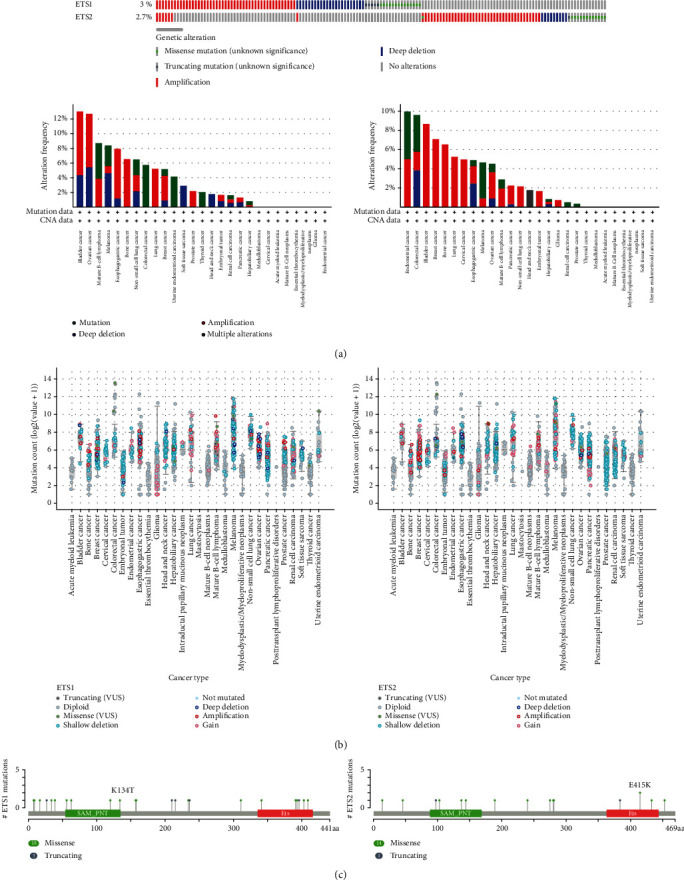
Mutation landscape of ETS1 and ETS2 in across cancers. (a) ETS1 and ETS2 mutation frequency across cancers. (b) The general mutation count of ETS1 and ETS2 across cancers. (c) Mutation diagram of ETS1 and ETS2 across protein domains.

The cBioPortal was applied to explore the mutation landscape of ETS1 and ETS2 across cancers. Alteration frequencies of ETS1 and ETS2 were both lower than 8% across cancers (Figure 6(a)). The top 5 cancers containing ETS1 alteration were BLCA, OV, mature B-cell lymphoma (MBL), melanoma, and ESCA. The top 5 types of cancer containing ETS2 alteration were UCEC, colorectal cancer, BLCA, BRAC, and bone cancer. Both mutation and amplification were the main type of alterations of ETS1 and ETS2. Deep deletion of ETS1 was more frequent than ETS2 (Figure 6(b)). A total of 18 missense and 5 truncating mutations were found in ETS1, while 14 missense and 1 truncating mutation were found in ETS2 (Figure 6(c)).

### 3.6. Enrichment Analysis of ETS1/ETS2-Related Partners

**Figure 7:**
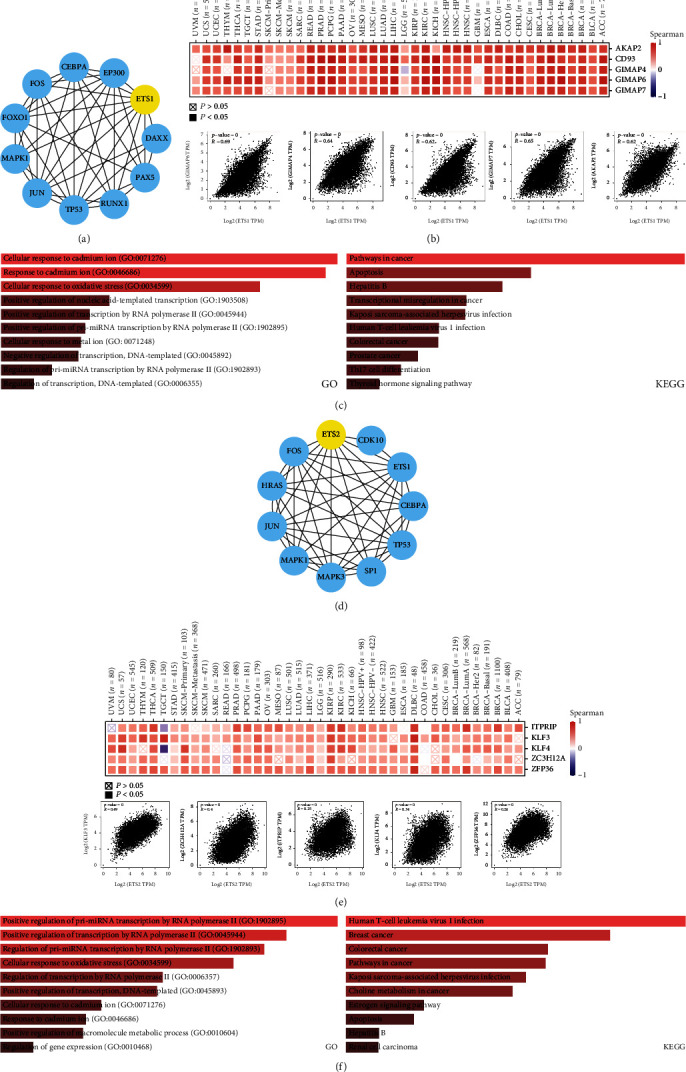
Enrichment analysis of ETS1/ETS2-related partners. (a) ETS1-binding proteins identified using STRING. (b) The top 5 ETS1-correlated genes across cancers were obtained from GEPIA 2. The upper panel showed the correlation between ETS1 and the top 5 genes across cancers, and the lower panel showed the correlation between ETS1 and the top 5 genes in overall TCGA patients. (c) GO and KEGG enrichment analysis of ETS1-binding and correlated genes. (d) ETS2-binding proteins identified using STRING. (e) The top 5 ETS2-correlated genes across cancers were obtained from GEPIA 2. The upper panel showed the correlation between ETS2 and the top 5 genes across cancers, and the lower panel showed the correlation between ETS2 and the top 5 genes in overall TCGA patients. (f) GO and KEGG enrichment analysis of ETS2-binding and correlated genes.

ETS1/ETS2 interacting and correlated genes were identified using STRING and GEPIA2. Ten ETS1 interacting genes were screened using STRING (Figure 7(a)). The top 5 ETS1 correlated genes were identified with GEPIA2, including GIMAP7, GIMAP6, GIMAP4, CD93, and AKAP2 (Figure 7(b)). Strong positive correlation between ETS1 and the top 5 correlated genes was observed across cancers. The GO enrichment analysis revealed enrichment in GO terms in response to cadmium ion and response to oxidative stress. KEGG analysis enrichment in pathways in cancer and apoptosis are shown in Figure 7(c).

Similarly, ten ETS2 interacting genes were screened using STRING (Figure 7(d)). The top 5 ETS2 correlated genes included ZFP36, ZC3H12A, KLF4, KLF3, and ITPRIP (Figure 7(e)). The GO enrichment analysis revealed enrichment in GO terms of transcription regulation. KEGG analysis enrichment in several types of cancer is shown in Figure 7(f).

## 4. Discussion

The RAS/MAPK pathway has been widely studied, and its constitutive activation has been observed frequently in a variety of cancers [14]. The polyomavirus enhancer region of the RAS/MAPK response sequence contains adjacent binding sites for ETS1 and ETS2 [15]. Moreover, ETS1/ETS2 cooperates with c-Jun and c-Fos to promote transcription from the polyomavirus enhancer region [16]. However, the prognostic value and immune features of ETS1 and ETS2 across cancers remain poorly understood.

The expression profile of ETS family in normal and tumor tissues was obtained from HPA and GEPIA2. ETS1 and ETS2 showed different tissue specificities in normal tissues. ETS1 was enriched in lymphoid tissues, while ETS2 showed low tissue specificity. On cellular level, ETS1 was enriched in NK, DC, and T cells, while ETS2 was enriched in basal squamous epithelial and prostatic cells. Pan-cancer analysis of the expression of ETS family showed that ETS1 was upregulated in 12 and downregulated in 6 types of cancers. However, ETS2 was upregulated in 4 and downregulated in 13 types of cancers. Both ETS1 and ETS2 were increased only in PAAD and STAD. On the other hand, both ETS1 and ETS2 were decreased in LUAD, LUSC, OV, UCEC, and UCS. These results reveal that ETS1 and ETS2 showed both similar and different expression patterns across cancers.

ETS1 and ETS2 showed different prognostic values across cancers. High expression of ETS1 and ETS2 was associated with longer OS in LIHC and KIRC. ETS1 level was associated with good prognosis in READ, BLCA, and BRCA, while associated with poor prognosis in KIRP, SARC, and PAAD. ETS2 was only negatively correlated with prognosis of OV, while positively correlated with prognosis of UCEC, EAC, and THYM.

Immune cells infiltration into the tumor microenvironment (TME) provides immunotherapy targets [17]. Generally, ETS1 showed stronger correlation with infiltrating immune cells compared with ETS2. ETS1 was positively correlated with several infiltrating immune cells across cancers. THYM showed unique correlation between ETS1 and immune cells. ETS1 showed negative correlation with B cells, macrophages/monocytes, CAFs, and NK cells, while positively correlated with DCs and T cells in THYM. Consistent with a previous study, ADA1 showed negative correlation with macrophages/monocytes, CAFs, and NK cells, while positively correlated with T cells and DCs in THYM [18]. These studies indicate unique TME other than other cancers. For ETS2, it was negatively correlated with T cells and B cells, while positively correlated with macrophages/monocytes, DCs, and CAFs in TGCT.

Immune checkpoints are immune regulators critical to maintain self-tolerance, magnitude, and duration of immune responses [19]. Immune checkpoint pathways prevent tumors from being recognized and killed by the immune system. Immune checkpoint blockade therapies applied antibodies targeting immune checkpoint pathways, including PD1, PD-L1, and CTLA-4 [20]. Similar with results from infiltrating immune cells correlation, ETS1 showed stronger correlation with several immune checkpoint genes in multiple cancers than ETS2. In THYM, ETS1 was negatively correlated with many immune checkpoints. THYM showed unique correlation between immune checkpoint genes and ETS1 expression compared with other types of cancers. ETS2 was negatively correlated with several immune checkpoint genes in some cancers, including COAD, LUAD, and LUSC.

Both ETS1 and ETS2 showed low mutation ratio (3% and 2.7%, respectively). It may be understandable because ETS1 and ETS2 were required for the survival of endothelial cells during embryonic angiogenesis. Mutation of ETS1 or ETS2 led to embryonic lethality [21], indicating their essential roles. Amplification and mutation were the top 2 alterations in ETS1 and ETS2. ETS1 harbored a higher ratio of deep deletion than ETS2 in several cancers. ETS1 and ETS2 carried similar number of missense or truncating mutations.

The ETS1/EST2 interacting and correlated genes were screened. The top 5 correlated genes showed positive correlation with ETS1/ETS2 in multiple cancers. KEGG enrichment analysis revealed enrichment in several cancers. GO enrichment showed that ETS1 interacting and correlated genes were enriched in GO terms in response to cadmium ion and response to oxidative stress, while ETS2 interacting and correlated genes were enriched in transcription regulation. Oxidative stress activated numerous transcription factors including p53, *β*-catenin/Wnt, Nrf2, HIF-1*α*, and PPAR-*γ*, resulting in the transcription of more than 500 genes, including cell cycle regulatory molecules, inflammatory cytokines, anti-inflammatory molecules, and growth factors, to promote the transformation of normal cells to tumors [22]. ETS1 overexpression led to reduced ROS along with increased expression of glutathione peroxidase, thereby improving the response to oxidative stress in ovarian and breast cancers [23]. Gene dysregulation is one of the hallmarks of cancer. Molecular regulators of transcription regulation are coming into view as novel attractive targets of drugs which perturb their functions and the transcriptional programs they governed [24].

In conclusion, we demonstrate the roles of ETS1 and ETS2 across cancers. ETS1 was increased in several cancers, while ETS2 was decreased in several cancers. Both ETS1 and ETS2 were favorable prognostic markers in LIHC and KIRC, while they showed different prognostic roles in more cancers. The different prognostic values for ETS1 and ETS2 may be partially explained by their different correlation with infiltrating immune cells and immune checkpoint gene. Moreover, ETS1 and ETS2 interacting and correlated genes were enriched in different GO terms. Thus, ETS1 and ETS2 play different roles in cancer development.

## Data Availability

The data used to support the findings of this study are included within the article.

## Abbreviations

ESCA: Esophageal carcinoma
DLBC: Lymphoid neoplasm diffuse large B-cell lymphoma
GBM: Glioblastoma multiforme
HNSC: Head and neck squamous cell carcinoma
LAML: Acute myeloid leukemia
KIRC: Kidney renal clear cell carcinoma
LGG: Brain lower grade glioma
SKCM: Skin cutaneous melanoma
PAAD: Pancreatic adenocarcinoma
STAD: Stomach adenocarcinoma
THYM: Thymoma
TGCT: Testicular germ cell tumors
LUAD: Lung adenocarcinoma
LUSC: Lung squamous cell carcinoma
CESC: Cervical squamous cell carcinoma and endocervical adenocarcinoma
UCEC: Uterine corpus endometrial carcinoma
OV: Ovarian serous cystadenocarcinoma
UCS: Uterine carcinosarcoma
COAD: Colon adenocarcinoma
READ: Rectum adenocarcinoma
BRCA: Breast invasive carcinoma
BLCA: Bladder urothelial carcinoma
KICH: Kidney chromophobe
THCA: Thyroid carcinoma
PRAD: Prostate adenocarcinoma
EAC: Esophageal adenocarcinoma
CAF: Cancer-associated fibroblast
TME: Tumor microenvironment.
